# Supplementary material for: Household characteristics associated with surface contamination of SARS-CoV-2 and frequency of RT-PCR and viral culture positivity–California and Colorado, 2021
Source: PLoS One. 2022 Oct 10;17(10):e0274946. doi: 10.1371/journal.pone.0274946 (PMC9550039; doi:10.1371/journal.pone.0274946)
Supplement: Attachment — Submitted filename: Response to reviewers.docx [file pone.0274946.s005.docx]

**Response to Reviewers**

**Household characteristics associated with surface contamination of SARS-CoV-2 and frequency of RT-PCR and viral culture positivity – California and Colorado, 2021.**

**Reviewer One**

1. Minor edits are suggested in track changes and the editor can go ahead with publishing while authors accept or reject these. I would strongly recommend an acknowledgement to those households who willingly participated in the study.

*All suggested edits have been reviewed and are acceptable to the authors. An acknowledgement to the participating households has been added on line 570-571.*

2. The reviewer was interested in the importance of the cleaning regime / timing (when last the house was cleaned) combined with size of households and number of individuals with symptoms. Correlation of these together with timing of testing with onset of illness would be interesting in future. If the main caregiver is ill and the household is not cleaned as regularly as always, it could possibly add to surface contamination.

*The authors appreciate this comment; we agree that analyzing the correlation between timing of last cleaning and positive surface samples would be interesting and useful. Unfortunately, our analysis is limited to the survey questions asked, and we did not include any questions about the timing of the last cleaning. However, the survey question “How frequently are you cleaning household surfaces” is in reference to current practices while enrolled in our study rather than general practices, which gives us some idea of how ongoing cleaning while household members are sick or lack there-of correlates to surface sample positivity. This is included as a footnote on Table 3 (“All household characteristics were evaluated at time of environmental sampling”), and we have additionally added this clarification to the methods section on line 155-156.*

3. One of the main questions / queries are the impact of asymptomatic carriers to the level of surface contamination. The reviewer is not clear if this has been dealt with in terms of the antigen test, however false negative test results should then be considered.

*We did not assess in detail the transmission from asymptomatic individuals on fomite surfaces; however, tables 3 and 4 indicate that households that were symptomatic had a statistically higher number of positive surface swabs detected by RT-PCR. Also, viable virus was isolated in only 3 swabs in our study. Taken together, our results indicate that fomite contamination is more likely to occur if individuals are symptomatic in a household but the risk of transmitting viable virus to a susceptible contact through fomites was very low.*

4. To clarify – was [environmental sampling] repeated? Was swabs taken at interim and again on day 14 within the same household once either at interim or on day 14? This could possibly explain some of the low positive test results.

*We have clarified in the text, and line 187-189 has been revised to read “In addition to swabbing at the initial visit, environmental sampling was offered to a subset of households at both interim and Day 14 closeout visits when sufficient swabs were available.”*

5. In reference to table 1, the reviewer has asked if we could include Household cleaning of surfaces as a table variable.

*This table is meant to summarize key household characteristics, not household behaviors. The distribution of households cleaning surfaces on a Likert scale is shown in table 3*

6. Was any of the individual household’s data further investigated and were there any interesting aspects (even though the sample size was too small to conclude anything worth reporting)? Specifically, the day of onset, the inclusion of children younger than 12 etc. Was this household different in terms of size, cleaning, etc.

Lines 409-419 have been revised to include additional detail:

*“The household had two members; the primary case was a 50-year-old female, and the secondary case was an 18-year-old male, with no other household members residing in this household. The primary case first tested positive five days before environmental surface sampling, and the secondary case first tested positive on the day of surface sampling. Both were symptomatic on day of sampling, each reporting eight of 15 symptoms surveyed, including nasal symptoms and cough. Neither reported diarrhea. The primary case reported a history of hypertension, hyperlipidemia, and unspecified immunosuppression. The household was a 3-bed 3-bathroom between 2000-3000 square feet. Household members reported sometimes using AC, using heating most of the time, and sometimes opening windows for ventilation and using fans. The household reported never cleaning high-touch household surfaces. The Epsilon variant was characterized from NP specimens of both household members.”*

**Reviewer Two**

This reviewer did not make any suggested revisions.
